# The significance of countable and treatable metastatic liver disease in patients with gallbladder carcinoma after curative‐intent surgery: A 10‐year experience in China

**DOI:** 10.1002/cam4.6450

**Published:** 2023-08-10

**Authors:** Tian‐Run Lv, Fei Liu, Wen‐Jie Ma, Hai‐Jie Hu, Yan‐Wen Jin, Fu‐Yu Li

**Affiliations:** ^1^ Department of General Surgery, Biliary Track Surgery West China Hospital of Sichuan University Chengdu China

**Keywords:** gallbladder carcinoma, liver metastasis, prognosis, surgery

## Abstract

**Objective:**

Our study was performed to evaluate the significance of countable and treatable metastatic liver disease (CTMLD) in patients with gallbladder carcinoma (GBC) after curative‐intent surgery.

**Methods:**

Resected GBC patients between September 2010 and January 2021 were reviewed. Comparative analyses between patients with CTMLD and those without it were performed. A propensity score matching analysis was conducted for further validation.

**Results:**

A total of 326 resected GBC patients were identified (33 with CTMLD). A significantly higher preoperative CA199 level was detected in those with CTMLD (*p* = 0.0160). Significantly higher incidences of major hepatectomy (*p* = 0.0010), lymph node metastasis (*p* < 0.0001), direct liver invasion (*p* < 0.0001), moderate to poor differentiation status (*p* < 0.0001), and T3–4 disease (*p* < 0.0001) were detected in patients with CTMLD. Even sharing comparable surgical margin status, patients with CTMLD still shared a significantly higher recurrence rate (93.9% vs. 57.3%, *p* < 0.0001) as well as a significantly higher recurrence rate within 6 months after surgery (63.6% vs. 14.7%, *p* < 0.0001). A significantly worse overall survival (median survival time: 12 vs. 33 months, *p* < 0.0001) or disease‐free survival (median recurrence‐free time: 6 vs. 30 months, *p* < 0.0001) was acquired in patients with CTMLD. After matching, a significantly higher early recurrence rate was still detected. Adjuvant chemotherapy seemed to have survival benefit for patients with CTMLD.

**Conclusion:**

CTMLD was an indicator of advanced disease and served as an independent predictor of early recurrence among resected GBC patients. Whether curative‐intent surgery is superior to nonsurgical treatment among GBC patients with CTMLD remains to be explored in future prospective studies.

## 1 INTRODUCTION

Gallbladder carcinoma (GBC) is a rare but fatal biliary epithelium‐originated malignancy.^1^ Due to lack of symptomatic clinical manifestations in the early phase of the disease, the majority of cases with GBC were diagnosed in an advanced stage and therefore lost the opportunity of receiving curative surgery.^1^ Simple cholecystectomy is especially effective in achieving tumor clearance among T1a GBC patients with a reported 5‐year survival rate reaching 100%.^2^ For patients with T1b or more advanced disease, the radical cholecystectomy, including simple cholecystectomy, partial hepatectomy, and a regional or more extended lymphadenectomy, has always been regarded as the gold standard in the management of patients with GBC. A significantly prolonged postsurgical survival period has been observed among resected GBC patients than those who failed to receive the radical cholecystectomy.^1^

**FIGURE 1:**
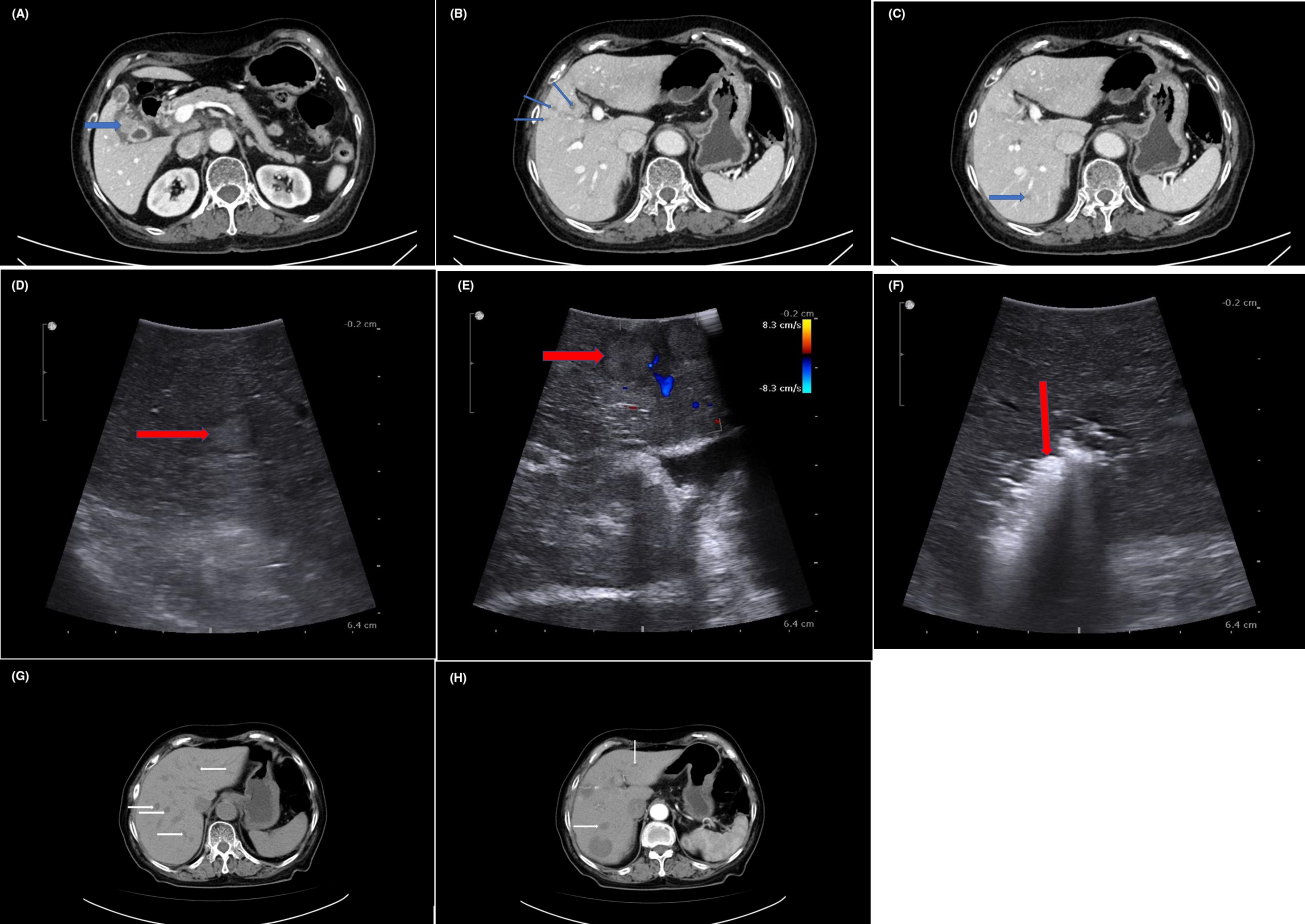
Representative figures of GBC patients with CTMLD. (A) primary gallbladder lesion (blue arrow); (B) regional metastatic liver lesions (blue arrow); (C) distant isolated metastatic liver lesion; (D) and (E) metastatic liver lesions detected by intraoperative ultrasound; (F) intraoperative radiofrequency ablation. The patient finally received a gallbladder excision, partial hepatectomy (SIVB + V), a regional lymphadenectomy, and a combined radiofrequency ablation (S7 metastatic lesion). Nearly 2 months after surgery, recurrent disease were detected within liver (G) and (H) (white arrow). CTMLD, countable and treatable metastatic liver disease; GBC, gallbladder carcinoma.

Tumor metastasis has long been considered as the major obstacle in the successful management of various malignancies and liver is one of the most common metastatic site among the majority of digestive malignancies.^3^ Great progress has been achieved in the treatment of patients with liver metastases of colorectal cancer and satisfactory long‐term survival has been reported after a combination of medical and surgical therapies,^4^ with a reported 5‐year survival rate reaching 58%.^5^ Liver has also been introduced as a frequently detected metastatic site of GBC.^6^, ^7^ According to the latest 8th American Joint Committee on Cancer (AJCC) staging criteria, the common metastatic sites of patients with GBC are peritoneum, liver, and occasionally to the lungs or pleura, which has been validated in previous researches that liver is the leading metastatic site and could be detected in over half of GBC patients.^6^, ^7^ Liver metastasis has been introduced as an indicator of advanced stage and predicted poor prognosis among GBC patients.^6^, ^7^ Over 90% GBC patients with liver dissemination were considered to be inoperable and were observed to succumb quickly due to advanced tumor‐related cachexia.^8^ However, clinically, GBC patients with liver metastasis can be furtherly divided into two subtypes, including operable and inoperable types. Inoperable type often indicated uncountable or diffusely disseminated metastatic tumor nodules within the liver, which could not be totally removed via invasive surgical procedures, such as partial hepatectomy or ultrasound‐guided percutaneous or intraoperative radiofrequency ablation (RFA). As for operable type, as presented in Figure 1, the number of metastatic lesions within the liver were countable and often concentrated in one segment or several adjacent liver segments (Figure 1A,B), which could be totally resected via a combined partial hepatectomy. Moreover, for cases with metastatic nodules located in a disseminated manner (Figure 1C), tumor clearance could also be achieved via a combination of RFA or minor hepatectomy. The countable and treatable metastatic liver disease (CTMLD) can be frequently detected among GBC patients but has been rarely reported. Currently, it has not reached a consensus on the optimal therapeutic regime for GBC patients with CTMLD. Besides, the prognostic significance of CTMLD among resected GBC patients has also been rarely explored. Whether CTMLD in patients with GBC should be regarded as an indicator of distant metastasis and therefore precludes curative surgery or not as an indicator of distant metastasis and curative surgery is feasible remains unknown. Furthermore, the effect of postoperative adjuvant chemotherapy has also been rarely explored among resected GBC patients with CTMLD. According to the latest National Comprehensive Cancer Network (NCCN) guidelines (www.nccn.org/guidelines/guidelines‐detail?category=1&id=1517), an up to 6‐month postoperative adjuvant chemotherapy or chemoradiotherapy was associated with survival benefit among resected GBC patients, especially in those with lymph node metastasis, which has also been validated in a recently reported phase 3 clinical trial that the efficiency of S‐1 based chemotherapy was revealed in biliary malignancies.^9^ However, whether postoperative adjuvant chemo or chemoradiotherapy would bring survival benefit for GBC patients with CTMLD after curative‐intent surgery remains unknown.

Therefore, based on our own single‐center experience, we aimed to explore the significance of CTMLD in patients with GBC after curative surgery in terms of tumor‐related clinicopathologic features and long‐term survival. A propensity score matching (PSM) was also performed for further validation.

## 2 METHODS

### 2.1 Patients

We retrospectively reviewed surgically treated GBC patients between September 2010 and January 2021 at West China Hospital, Sichuan University. Comparative analyses were performed to evaluate the prognostic significance of CTMLD in resected GBC patients and therefore the postoperative long‐term survival is the primary endpoint of our study. In order to control various confounding factors which might lead to unpredictable survival influence to our results, patients who died within 6 months after surgery were excluded in order to guarantee the quality of our cohort. This study was approved by institutional ethics review board of West China Hospital (approval number: ChiCTR2300071754).

### 2.2 The definition of CTMLD

CTMLD was defined as follows:

1. Regardless of direct liver invasion, an isolated metastatic liver nodule was detected radiologically in any lobe or segment within the whole liver rather than the primary site, which could be removed combined with minor hepatectomy or RFA.
2. Regardless of direct liver invasion, numerous countable metastatic nodules were detected within one segment of the liver or within two or three adjacent segments, which can be totally removed combined with partial hepatectomy or RFA.
3. Regardless of direct liver invasion, numerous countable metastatic nodules were detected in nonadjacent liver segments and these lesions can be furtherly removed combined with partial hepatectomy and RFA.

### 2.3 Preoperative management and surgery

Upon admission, systematic body examinations, including blood test and radiological imaging examinations like computed tomography (CT) or magnetic resonance image (MRI) or even PET–CT, were applied in all surgical candidates to have an initial evaluation of the tumor stage as well as to exclude patients who were contraindicated to curative‐intent surgery. Surgery candidates should fulfill the following criteria. First, patients with serious comorbidities, such as stroke or sever coagulation abnormalities, were not allowed. Second, an estimated functional liver remnant volume >40% after partial hepatectomy was always required. Third, cases with radiologically suspected distant metastasis (abdominal dissemination, para‐aortic lymph node enhancement, or lung metastasis) were not allowed. All surgical candidates would receive a multidisciplinary discussion to find the optimal therapeutic regime. For patients with CTMLD, their families were informed of their disease status that liver metastasis has already occurred and the effect of curative‐intent surgery might be less satisfactory than those without CTMLD. If they still insisted on performing curative‐intent surgery, they were required to sign the surgery‐related informed consent and therefore surgery would be performed at the premise of their strong desire and written request. The specific surgical procedures were in consistent with our recently published series^10^ and partial hepatectomy combined with or without RFA would be performed in order to achieve tumor clearance. All patients were staged based on the 8th AJCC staging system.

### 2.4 Follow‐up

Postoperative patients reexamined their disease status at the outpatient of our hospital every 1–2 months in the first 2 years after surgery and every 6 months thereafter. Their survival status was updated once a year via telephone number recorded in their electronic database. The most recent update was performed on September 1, 2022.

### 2.5 Statistical analysis

IBM SPSS version 25.0 and Graph‐Pad Prism 7 were used for statistical analysis. Categorical data were presented as numbers (percentages). Categorical variables were compared through chi‐squared or Fisher's exact tests. overall survival (OS) indicated the survival period from the date of surgery to the date of death or last follow‐up. Disease‐free survival (DFS) indicated the survival time from the date of surgery to the date of radiologically confirmed disease recurrence. Kaplan–Meier curves were applied in depicting survival outcomes and log‐rank test was used in evaluating survival differences. Univariate and multivariate Cox regression model were used to evaluate potential prognostic factors and independent prognostic factors for OS and DFS of the entire cohort. Results were recorded as hazard ratio (HR) with its 95% confidence interval (CI). A *p* value < 0.05 indicated statistical difference. Binary logistic regression was furtherly applied to identify the potential predictors of early recurrence. A PSM was also performed to control bias and for further validation (ratio 1:1, standard deviation 0.1).

## 3 RESULTS

### 3.1 Baseline characteristics of the entire cohort

**FIGURE 2:**
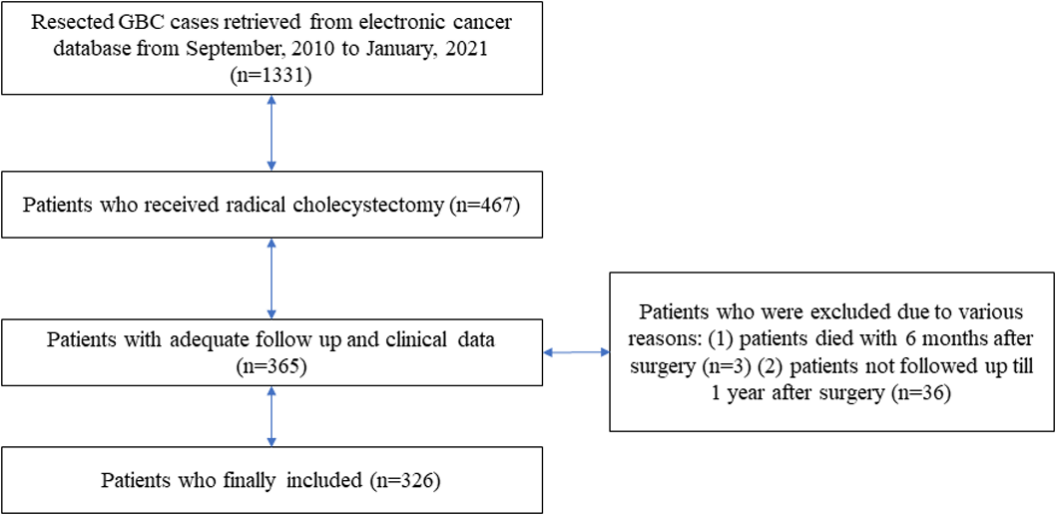
Flowchart of the specific process of patient selection and identification. GBC, gallbladder carcinoma.

Initially, a total of 329 resected GBC patients with adequate follow‐up and clinical data were identified. In order to guarantee the quality of our cohort as previously descripted, three cases who died within 6 months after surgery were excluded. Two cases died perioperatively due to the severe abdominal hemorrhage derived from the spontaneous rupture of pseudoaneurysm. Another patient died 3 months after surgery due to unpredicted and explosive liver failure after major hepatectomy. Finally, 326 resected GBC patients were included in our study, 33 (10.1%) of whom were diagnosed with CTMLD. The specific process of patient selection and identification was presented in Figure 2. The overall sex ratio was nearly 2:1 (female: male, 221: 105). The majority of cases were diagnosed order than 60 (171, 52.5%). Forty patients (12.3%) were presented with obstructive jaundice preoperatively and six (1.8%) of them received preoperative biliary drainage (PTCD or ENBD). Sixty‐four (19.6%) patients received combined extra‐hepatic bile duct resection, 35 patients (10.7%) received major hepatectomy, 39 (12.0%) patients received combined multi‐visceral resection, and 14 (4.3%) patients received combined major vascular resection and reconstruction. The overall incidence of lymph node metastasis was 38% (124/326) and the overall incidence of direct liver invasion was 32.6% (142/326). The overall R0 rate was 91.4% (298/326). Eighty‐six patients have received at least four cycles of postoperative intravenous‐mediated adjuvant systematic chemotherapy. Two chemotherapy agents, Gemcitabine plus Cisplatin, served as the corner stone of the postoperative chemotherapy. The median survival time of the entire cohort was 28.5 months, ranging from 6 to 124 months.

### 3.2 Comparative analysis between patients with CTMLD and those without CTMLD before PSM

**FIGURE 3:**
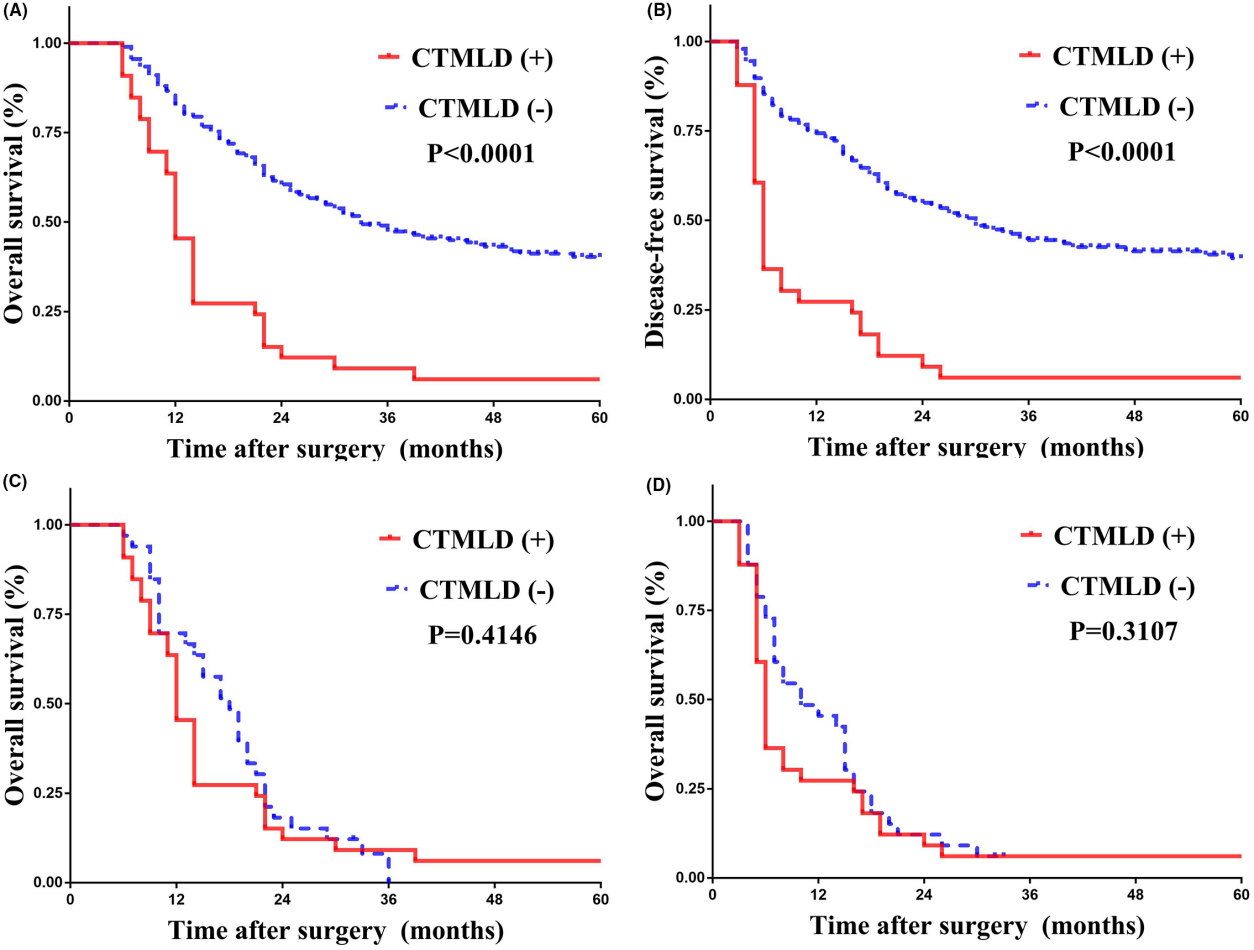
KM curves presenting survival outcomes before and after PSM. (A) OS before PSM; (B) DFS before PSM; (C) OS after PSM; (D) DFS after PSM. CTMLD, countable and treatable metastatic liver disease; DFS, disease‐free survival; OS, overall survival; PSM, propensity score matching.

As is summarized in Table 1, patients with CTMLD shared comparable sex ratio and age status versus patients without CTMLD. However, a significantly higher percentage of patients with preoperative CA199 level >37 U/mL was detected in those with CTMLD (54.5% vs. 33.4%, *p* = 0.0160). Moreover, significantly higher incidences of major hepatectomy (27.3% vs. 8.9%, *p* = 0.0010), lymph node metastasis (75.8% vs. 33.8%, *p* < 0.0001), direct liver invasion (87.9% vs. 38.2%, *p* < 0.0001), moderate to poor differentiation status (100.0% vs. 77.8%, *p* < 0.0001), and T3–4 disease (97.0% vs. 60.1%, *p* < 0.0001) were detected in patients with CTMLD. Even though patients with CTMLD and those without CTMLD shared a comparable incidence of negative margin, patients with CTMLD shared a significantly higher recurrence rate (93.9% vs. 57.3%, *p* < 0.0001) as well as a significantly higher recurrence rate within 6 months after surgery (63.6% vs. 14.7%, *p* < 0.0001). Survival analyses indicated that patients with CTMLD shared a much worse OS (median survival time: 12 vs. 33 months, *p* < 0.0001) (Figure 3A) as well as DFS than those without CTMLD after surgery (median recurrence‐free time: 6 vs. 30 months, *p* < 0.0001; Figure 3B).

**TABLE 1:** Baseline characteristics of the entire cohort before PSM.

Variables	Patients with CTMLD (*n* = 33)	Patients without CTMLD (*n* = 293)	*p*‐Value
Sex			
Male	12 (36.4%)	93 (31.7%)	0.5900
Female	21 (63.6%)	200 (68.3%)	
Age			
≥60	14 (42.4%)	157 (53.6%)	0.2240
<60	19 (57.6%)	136 (46.4%)	
Preoperative CA199			
≥37 U/mL	18 (54.5%)	98 (33.4%)	0.0160
<37 U/mL	15 (45.5%)	195 (66.6%)	
Obstructive jaundice			
Yes	3 (9.1%)	37 (12.6%)	0.7590
No	30 (90.9%)	256 (87.4%)	
Preoperative biliary drainage			
Yes	0 (0.0%)	6 (2.0%)	0.5240
No	33 (100.0%)	287 (98.0%)	
Bile duct resection			
Yes	4 (12.1%)	60 (20.5%)	0.3600
No	29 (87.9%)	233 (79.5%)	
Major hepatectomy^a^			
Performed	9 (27.3%)	26 (8.9%)	0.0010
Not performed	24 (72.7%)	267 (91.1%)	
Combined multi‐visceral resections^c^			
Performed	3 (9.1%)	36 (12.3%)	0.0800
Not performed	30 (90.9%)	257 (87.7%)	
Major vascular resection and reconstruction^b^			
Performed	3 (9.1%)	11 (3.8%)	0.3270
Not performed	30 (90.9%)	282 (96.2%)	
Surgical margin			
Negative	29 (87.8%)	269 (91.8%)	0.6630
Positive	4 (12.2%)	24 (8.2%)	
Lymph node metastasis			
Yes	25 (75.8%)	99 (33.8%)	<0.0001
No	8 (24.2%)	194 (66.2%)	
Perineural invasion			
Yes	7 (21.2%)	59 (20.1%)	0.8840
No	26 (78.8%)	234 (79.9%)	
Lymph‐vascular invasion			
Yes	7 (21.2%)	37 (12.6%)	0.1710
No	26 (78.8%)	256 (87.4%)	
Liver invasion			
Yes	29 (87.8%)	112 (38.2%)	<0.0001
No	4 (12.2%)	181 (61.8%)	
Differentiation status			
Poor to moderate	33 (100.0%)	228 (77.8%)	<0.0001
High	0 (0.0%)	65 (22.2%)	
T stage			
T1–T2	1 (3.0%)	117 (39.9%)	<0.0001
T3–T4	32 (97.0%)	176 (60.1%)	
Pathology (AC)			
Yes	5 (15.2%)	28 (9.6%)	0.3120
No	28 (84.8%)	265 (90.4%)	
Morbidities^d^			
Yes	5 (15.2%)	45 (15.4%)	0.9750
No	28 (84.8%)	248 (84.6%)	
Postoperative chemotherapy			
Performed	4 (12.1%)	82 (28.0%)	0.0800
Not performed	29 (87.9%)	211 (72.0%)	
Recurrence			
Yes	31 (93.9%)	168 (57.3%)	<0.0001
No	2 (6.1%)	125 (42.7%)	
Recurrence within 6 months after surgery			
Yes	21 (63.6%)	43 (14.7%)	<0.0001
No	12 (36.4%)	250 (85.3%)	

Abbreviations: AC, adenocarcinoma; CTMLD, countable and treatable metastatic liver disease; PSM, propensity score matching.

^a^ Resected more than four segments.

^b^ Portal vein, hepatic artery or hepatic vein.

^c^ Stomach, colon, small intestine, or pancreas; PSM.

^d^ Hepatic failure, lung infection, hemorrhage, peritoneal cavity infection, pancreatic fistula, bile leakage, sepsis, renal failure, and acute cardiac failure.

### 3.3 Univariate and multivariate analyses of prognostic factors for OS and DFS

As is summarized in Table 2, serum preoperative CA199 (<37 U/mL vs. ≥37 U/mL) (*p* < 0.0001), lymph node metastasis (−vs. +) (*p* < 0.0001), perineural invasion (−vs. +) (*p* < 0.0001), lymph‐vascular invasion status (−vs. +) (*p* < 0.0001), liver invasion (−vs. +) (*p* < 0.0001), surgical margin status (negative vs. positive) (*p* < 0.0001), tumor differentiation status (well vs. poor or moderate) (*p* < 0.0001), T stage (T1–2 vs. T3–4) (*p* < 0.0001), CTMLD (− vs. +) (*p* < 0.0001), pathology (others vs. adenocarcinoma) (*p* = 0.0030), and postoperative chemotherapy (not performed vs. performed) (*p* < 0.0001) were prognostic factors for OS. Results of multivariate analysis indicated that preoperative CA199 (<37 U/mL vs. ≥37 U/mL) (*p* = 0.0170), lymph node metastasis (− vs. +) (*p* < 0.0001), surgical margin status (negative vs. positive) (*p* < 0.0001), T stage (T1–2 vs. T3–4) (*p* < 0.0001), CTMLD (− vs. +) (*p* = 0.0050), and postoperative chemotherapy (− vs. +) (*p* < 0.0001) were independent prognostic factors for OS.

**TABLE 2:** Univariate and multivariate analysis of overall survival and DFS of all patients.

Variables	Univariate analysis			Multivariate analysis		
	HR	95% CI	*p*‐Value	HR	95% CI	*p*‐Value
Overall survival						
Sex (female vs. male)	0.922	0.683–1.245	0.5960			
Age (<60 vs. ≥60)	1.062	0.911–1.237	0.4430			
Preoperative CA199 (<37 U/mL vs. ≥37 U/mL)	3.139	2.356–4.182	<0.0001	1.486	1.073–2.057	0.0170
Node metastasis (− vs. +)	4.106	3.058–5.514	<0.0001	1.879	1.354–2.608	<0.0001
Perineural invasion (− vs. +)	2.599	1.891–3.574	<0.0001	1.301	0.910–1.859	0.1490
Lymph‐vascular invasion (− vs. +)	2.300	1.595–3.316	<0.0001	1.349	0.912–1.996	0.1330
Liver invasion (− vs. +)	4.051	3.007–5.457	<0.0001	1.002	0.691–1.454	0.9910
Surgical margin (negative vs. positive)	3.118	2.035–4.777	<0.0001	2.549	1.592–4.081	<0.0001
Tumor differentiation status (well vs. poor/moderate)	2.660	1.718–4.115	<0.0001	0.819	0.508–1.319	0.4110
T stage (T1–2 vs. T3–4)	9.186	5.885–14.338	<0.0001	5.192	3.024–8.914	<0.0001
CTMLD (− vs. +)	3.476	2.351–5.141	<0.0001	1.809	1.191–2.747	0.0050
Pathology (others vs. AC)	0.528	0.347–0.804	0.0030	0.930	0.575–1.504	0.7680
Postoperative chemotherapy (not performed vs. performed)	0.534	0.375–0.759	<0.0001	0.458	0.316–0.665	<0.0001
DFS						
Sex (female vs. male)	0.867	0.646–1.163	0.3410			
Age (<60 vs. ≥60)	1.045	0.892–1.224	0.5870			
Preoperative CA199 (<37 U/mL vs. ≥37 U/mL)	3.042	2.289–4.043	<0.0001	1.345	0.976–1.852	0.0700
Node metastasis (− vs. +)	3.928	2.938–5.252	<0.0001	1.852	1.344–2.552	<0.0001
Perineural invasion (− vs. +)	2.793	2.036–3.833	<0.0001	1.617	1.141–2.293	0.0070
Lymph‐vascular invasion (− vs. +)	2.074	1.428–3.012	<0.0001	1.230	0.829–1.825	0.3040
Liver invasion (− vs. +)	3.882	2.892–5.211	<0.0001	0.932	0.648–1.342	0.7060
Surgical margin (negative vs. positive)	3.413	2.225–5.235	<0.0001	2.819	1.740–4.566	<0.0001
Tumor differentiation status (well vs. poor/moderate)	2.488	1.634–3.788	<0.0001	0.837	0.530–1.321	0.4450
T stage (T1–2 vs. T3–4)	9.708	6.224–15.142	<0.0001	6.043	3.547–10.297	<0.0001
CTMLD (− vs. +)	3.634	2.455–5.379	<0.0001	1.906	1.253–2.898	0.0030
Pathology (others vs. AC)	0.537	0.358–0.807	0.0030	0.916	0.577–1.453	0.7090
Postoperative chemotherapy (not performed vs. performed)	0.574	0.408–0.807	0.0010	0.456	0.318–0.654	<0.0001

Abbreviations: AC, adenocarcinoma; CI, confidence interval; CTMLD, countable and treatable metastatic liver disease; DFS, disease‐free survival; HR, hazard ratio.

Serum preoperative CA199 (<37 U/mL vs. ≥37 U/mL) (*p* < 0.0001), lymph node metastasis (− vs. +) (*p* < 0.0001), perineural invasion (− vs. +) (*p* < 0.0001), lymph‐vascular invasion (− vs. +) (*p* < 0.0001), direct liver invasion (− vs. +) (*p* < 0.0001), surgical margin status (negative vs. positive) (*p* < 0.0001), tumor differentiation status (well vs. poor/moderate) (*p* < 0.0001), T stage (T1–2 vs. T3–4) (*p* < 0.0001), CTMLD (− vs. +) (*p* < 0.0001), pathology (others vs. adenocarcinoma) (*p* = 0.0030), and postoperative chemotherapy (not performed vs. performed) (*p* = 0.0010) were prognostic factors for DFS. Results of multivariate analysis indicated that lymph node metastasis (− vs. +) (*p* < 0.0001), Perineural invasion (− vs. +) (*p* = 0.0070), surgical margin status (negative vs. positive) (*p* < 0.0001), T stage (T1–2 vs. T3–4) (*p* < 0.0001), CTMLD (− vs. +) (*p* = 0.0030), and postoperative chemotherapy (not performed vs. performed) (*p* < 0.0001) were independent prognostic factors for DFS (Table 2).

### 3.4 Propensity score matching analysis

Owing to the retrospective nature of our cohort, many uncontrolled factors hindered us from further exploration on the significance of CTMLD in resected patients with GBC. Age, sex, and numerous independent prognostic factors (lymph node metastasis, T stage, CA199, postoperative chemotherapy, surgical margin) were controlled between CTMLD (+) and CTMLD (−) groups. After PSM, as is summarized in Table 3, a total of 66 patients were identified (ratio 1:1). Patients of two groups were comparable in all these matched factors. However, the incidence of moderate to poor differentiation status (100.0% vs. 84.8%, *p* = 0.0270) were still significantly higher in patients with CTMLD. Although comparable OS (*p* = 0.4146) (Figure 3C) and DFS (*p* = 0.3107) (Figure 3D) were acquired between two groups, a significantly higher recurrence rate within 6 months after surgery was still detected in patients with CTMLD (63.6% vs. 27.3%, *p* = 0.0030).

**TABLE 3:** Baseline characteristics of the entire cohort after PSM.

Variables	Patients with CTMLD (*n* = 33)	Patients without CTMLD (*n* = 33)	*p*‐Value
Sex			
Male	12 36.4%)	13 (39.4%)	0.8000
Female	21 (63.6%)	20 (60.6%)	
Age			
≥60	14 (42.4%)	15 (45.5%)	0.8040
<60	19 (57.6%)	18 (54.5%)	
Preoperative CA199			
≥37 U/mL	18 (54.5%)	17 (51.5%)	0.8050
<37 U/mL	15 (45.5%)	16 (48.5%)	
Obstructive jaundice			
Yes	3 (9.1%)	6 (18.2%)	0.4730
No	30 (90.9%)	27 (81.8%)	
Preoperative biliary drainage			
Yes	0 (0.0%)	0 (0.0%)	——
No	33 (100.0%)	33 (100.0%)	
Bile duct resection			
Yes	4 (12.1%)	13 (39.4%)	0.0240
No	29 (87.9%)	20 (60.6%)	
Major hepatectomy^a^			
Performed	9 (27.3%)	6 (18.2%)	0.3780
Not performed	24 (72.7%)	27 (81.8%)	
Combined multi‐visceral resections^c^			
Performed	3 (9.1%)	9 (27.3%)	0.1110
Not performed	30 (90.9%)	24 (72.7%)	
Major vascular resection and reconstruction^b^			
Performed	3 (9.1%)	2 (6.1%)	1.0000
Not performed	30 (90.9%)	31 (93.9%)	
Surgical margin			
Negative	29 (87.9%)	28 (84.8%)	1.0000
Positive	4 (12.1%)	5 (15.2%)	
Lymph node metastasis			
Yes	25 (75.8%)	25 (75.8%)	1.0000
No	8 (24.2%)	8 (24.2%)	
Perineural invasion			
Yes	7 (21.2%)	14 (42.4%)	0.0640
No	26 (78.8%)	19 (57.6%)	
Lymph‐vascular invasion			
Yes	7 (21.2%)	8 (24.2%)	0.7690
No	26 (78.8%)	25 (75.8%)	
Liver invasion			
Yes	29 (87.9%)	22 (66.7%)	0.0780
No	4 (12.1%)	11 (33.3%)	
Differentiation status			
Poor to moderate	33 (100.0%)	28 (84.8%)	0.0270
High	0 (0.0%)	5 (15.2%)	
T stage			
T1–T2	1 (3.0%)	1 (3.0%)	1.0000
T3–T4	32 (97.0%)	32 (97.0%)	
Pathology (AC)			
Yes	5 (15.2%)	5 (15.2%)	1.0000
No	28 (84.8%)	28 (84.8%)	
Morbidities^d^			
Yes	5 (15.2%)	10 (30.3%)	0.1420
No	28 (84.8%)	23 (69.7%)	
Postoperative chemotherapy			
Performed	4 (12.1%)	3 (9.1%)	1.0000
Not performed	29 (87.9%)	30 (90.9%)	
Recurrence			
Yes	2 (6.1%)	2 (6.1%)	1.0000
No	31 (93.9%)	31 (93.9%)	
Recurrence within 6 months after surgery			
Yes	21 (63.6%)	9 (27.3%)	0.0030
No	12 36.4%)	24 (72.7%)	

Abbreviations: AC, adenocarcinoma; CTMLD, countable and treatable metastatic liver disease; PSM, propensity score matching.

^a^ Resected more than four segments.

^b^ Portal vein, hepatic artery or hepatic vein.

^c^ Stomach, colon, small intestine, or pancreas; PSM.

^d^ Hepatic failure, lung infection, hemorrhage, peritoneal cavity infection, pancreatic fistula, bile leakage, sepsis, renal failure, and acute cardiac failure.

### 3.5 Logistic regression on predictors of early recurrence within 6 months

As is summarized in Table 4, serum preoperative CA199 (<37 U/mL vs. ≥37 U/mL), lymph node metastasis status (− vs. +), perineural invasion (− vs. +), surgical margin status (negative vs. positive), tumor differentiation status (well vs. poor/moderate), T stage (T1–2 vs. T3–4), CTMLD (+ vs. −), pathology (others vs. adenocarcinoma), and postoperative chemotherapy (not performed vs. performed) were predictors of early recurrence. However, results of multivariate analysis indicated that lymph node metastasis (− vs. +) (*p* = 0.0170), surgical margin status (negative vs. positive) (*p* < 0.0001), tumor differentiation status (well vs. poor/moderate) (*p* = 0.0420), CTMLD (− vs. +) (*p* < 0.0001), pathology (others vs. adenocarcinoma) (*p* = 0.0160), and postoperative chemotherapy (not performed vs. performed) (*p* = 0.0040) were independent predictors of early recurrence.

**TABLE 4:** Logistic regression on the potential predictors of early recurrence for patients with GBC.

Variables	*p‐*Value of univariate analysis	Multivariate analysis		
		OR	95% CI	*p*‐Value
Sex (male vs. female)	0.4770			
Age (<60 vs. ≥60)	0.3600			
Preoperative CA199 (<37 U/mL vs. ≥37 U/mL)	<0.0001	1.672	0.803–3.482	0.1700
Node metastasis (− vs. +)	<0.0001	2.709	1.191–6.162	0.0170
Perineural invasion (− vs. +)	<0.0001	1.664	0.736–3.762	0.2210
Surgical margin (negative vs. positive)	<0.0001	11.480	3.434–38.385	<0.0001
Tumor differentiation status (poor/moderate vs. well)	0.0020	0.141	0.021–0.934	0.0420
T stage (T1–T2 vs. T3–T4)	<0.0001	4.295	0.844–21.861	0.0790
CTMLD (− vs. +)	<0.0001	5.261	2.152–12.862	<0.0001
Pathology (others vs. AC)	0.0130	0.269	0.093–0.783	0.0160
Postoperative chemotherapy (not performed vs. performed)	0.0010	0.189	0.060–0.594	0.0040

Abbreviations: AC, adenocarcinoma; CI, confidence interval; CTMLD, countable and treatable metastatic liver disease; GBC, gallbladder carcinoma; OR, odds ratio.

### 3.6 The effect of postsurgical adjuvant chemotherapy in patients with CTMLD

**FIGURE 4:**
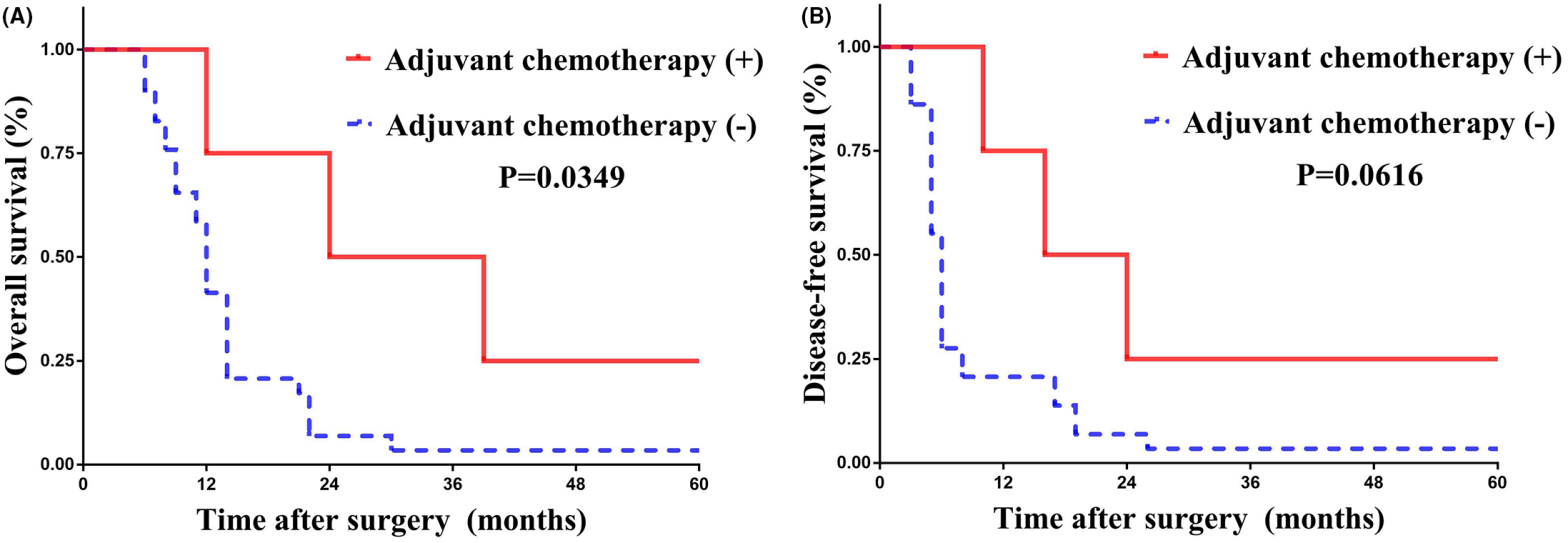
KM curves presenting survival outcomes between patients with CTMLD with adjuvant chemotherapy and those without adjuvant chemotherapy. (A) OS; (B) DFS. CTMLD, countable and treatable metastatic liver disease; DFS, disease‐free survival; OS, overall survival.

We subsequently explored the efficiency of postoperative adjuvant systematic chemotherapy in patients with CTMLD. Adjuvant chemotherapy was associate with a more favorable OS (median survival time: 31.5 vs. 12 months, *p* = 0.0349) among resected GBC patients with CTMLD (Figure 4A). Moreover, a more favorable DFS with a borderline *p* value (median survival time: 20 vs. 6 months, *p* = 0.0616) was also observed in patients with CTMLD who received adjuvant chemotherapy (Figure 4B).

## 4 DISCUSSION

To our knowledge, few researchers insisted on performing curative‐intent surgery among GBC patients with liver metastasis. Systematic chemotherapy, radiotherapy, RFA, targeted therapy, or other less invasive therapeutic regimes are clinically preferred in GBC patients with metastatic liver disease.^11^ However, we cannot deny the fact that for some experienced and aggressive centers, the curative surgery has been attempted to be applied in a small portion of patients with liver metastasis, especially among those with CTMLD. With the development of intraoperative ultrasound technique and the prevalence of RFA, partial hepatectomy combined with intraoperative ultrasound‐guided RFA has been extensively applied in various primary or secondary metastatic liver malignancies.^12^, ^13^, ^14^, ^15^ Our final results indicated that although GBC patients with CTMLD seemed to be operable, significantly worse OS and DFS were detected even patients were actively treated. Moreover, patients with CTMLD still shared a much higher early recurrence rate even after various independent prognostic factors were matched between two groups. CTMLD was an independent predictor of early recurrence. However, postoperative adjuvant chemotherapy seemed to have significantly prolonged the survival period of patients with CTMLD after curative surgery, which might provide clinical reference on the indication of postoperative adjuvant chemotherapy in GBC patients to some extent.

Liver metastasis has been a long‐standing but debating issue which greatly hindered the successful management of various cancers, particularly for cancers with great potential to develop metastatic liver disease, such as pancreatic cancer.^16^ However, with the evolution of hepatectomy‐related surgical techniques, ultrasound‐guided RFA, as well as progress in perioperative management, liver has never been considered as the restricted area in the surgical management of various primary or metastatic liver malignancies. RFA has been widely used as a minimally invasive surgical procedure for patients with primary or secondary liver malignancies. Ultrasound‐guided RFA combined with experienced manipulators could achieve similar oncological outcomes and long‐term survival as conventional partial hepatectomy in cases with small liver tumors, avoiding unnecessary surgery‐related trauma to human body, which has been validated by Zheng et al.^17^ Moreover, due to the survival benefit brought by adjuvant systematic chemotherapy, the prognosis of patients with colorectal metastatic liver disease has greatly improved, with a reported 5‐year survival rate reaching 60%.^5^ However, as for GBC patients with liver metastasis, their prospect still seems to be vague.

Relevant studies regarding GBC patients with liver metastasis are quite rare either clinically or basically, which might be partially due to the rarity of the disease. According to the latest 8th AJCC staging criteria, liver metastasis was just mentioned as an indicator of distant metastasis but was ill‐defined. In the latest NCCN guidelines, it has also not reached a consensus regarding the optimal therapeutic regime for GBC patients with metastatic liver disease, especially among those with CTMLD. Whether GBC patients with CTMLD should be treated aggressively via surgical procedures or treated conservatively via nonsurgical procedures remains unexplored. In order to supply the treatment reference, we have attempted to compare the overall prognosis between patients with CTMLD who received surgery and those who received nonsurgical treatment. However, having rescreened our cancer database, almost all patients with CTMLD have received curative‐intent surgery and the intended comparison was not feasible. Therefore, the absence of comparison between patients with CTMLD who received curative‐intent surgery and patients with CTMLD who received nonsurgical treatment might be a major limitation within our paper, which was expected to be explored in future prospective studies.

Clinically, in the most Asian centers, especially Japanese centers, surgeons have always been aggressive in the management of biliary malignancies.^18^, ^19^ Curative surgery has always been the most effective therapeutic modality in the majority of cases with digestive malignancies, even in those with advanced disease, which could greatly decrease tumor burden and has been actively pursued.^19^, ^20^, ^21^ For example, one previous meta‐analysis has systematically evaluated the significance of hepatopancreatoduodenectomy among GBC patients with advanced T4 disease and revealed that although high rates of morbidities and mortalities were detected, long‐term survival could also be achieved among patients for whom curative‐intent surgery were feasible.^22^ Moreover, from our own center's experience, surgeons' decisions on the optimal therapeutic regime of GBC with liver metastasis were mostly driven by the operability of the metastatic tumor. In other words, more specifically, whether the surgery would be performed or not was mainly decided by the clinicians' subjective judgment. If there are too many metastatic nodules within the liver or there are extensive abdominal dissemination, tumor clearance is often unfeasible via invasive surgical procedures. In this case, curative surgery would not be performed and systematic chemotherapy, chemoradiotherapy or targeted therapy would be preferred.^23^, ^24^ However, at least in our center, clinicians were more likely to perform invasive surgical procedures for GBC patients with CTMLD. Unfortunately, although these patients with CTMLD were actively treated and tumor clearance seemed to have been achieved via invasive surgery, patients with CTMLD still shared a worse prognosis even when tumor stage was matched. Moreover, even various vital prognostic factors have been matched, patients with CTMLD still shared more aggressive tumor biological features, including more awful tumor differentiation status, a higher frequency of liver invasion, and a significantly higher early recurrence rate. Therefore, CTMLD was a signal of advanced disease and patients with CTMLD were often presented with extensive metastatic dissemination, that is, the disease has evolved into a systematic disease rather than an isolated disease. This might partially explain the worse survival even being actively treated as well as the high recurrence rate after matching. Additionally, the treatment received after recurrence been detected might also influence the overall prognosis to some extent. The overall recurrence rate was 61.0% in our cohort and the majority of cases were presented with obstructive jaundice upon recurrence, combined with multifocal spread identified in the contrast‐enhanced CT scan. In their electronic medical record, most cases with recurrence only received palliative treatment, such as PTCD for patients with biliary obstruction and jejunal nutrition tube for patients with ileus. A few patients received palliative biliary‐enteric anastomosis or gastrointestinal anastomosis. Only a limited number of patients with favorable health conditions received systematic chemotherapy or targeted therapy. However, only 3–4 months extension of survival period was detected and the majority of cases with recurrence died within 6 months.

Another major finding of our study is the survival benefit brought by postoperative adjuvant chemotherapy in GBC patients with CTMLD. Accumulating evidence has demonstrated the survival benefit brought by postoperative adjuvant chemotherapy among resected biliary cancers.^9^, ^25^, ^26^ In consistent with previous findings, our data also indicated that postoperative adjuvant chemotherapy was a favorable prognostic factor with an obviously improved postoperative survival versus those who did not receive systematic adjuvant chemotherapy. Adjuvant systematic chemotherapy served as an independent prognostic factor for both OS and DFS. Furthermore, among patients with CTMLD, a significantly prolonged survival has also been detected after the application of adjuvant chemotherapy. Currently, as previously described, the application of adjuvant postoperative chemotherapy was especially recommended in GBC patients with lymph node metastasis. CTMLD might also be an indicator of adjuvant chemotherapy if an extended survival was observed among patients with CTMLD who received curative surgery than those who received nonsurgical treatment.

## 5 CONCLUSION

Current study first explored the significance of CTMLD among GBC patients after curative‐intent surgery. Even tumor stage was matched between two groups, patients with CTMLD still shared more aggressive tumor biological features and developed recurrences much earlier than those without CTMLD after curative‐intent surgery. CTMLD could be regarded as an indicator of advanced disease as well as an independent predictor of early recurrence for patients with GBC after curative‐intent surgery. Future prospective well‐designed studies are demanded for further exploration on the efficiency of curative‐intent surgery versus nonsurgical treatment among GBC patients with CTMLD.

## AUTHOR CONTRIBUTIONS

**Tian‐Run Lv:** Conceptualization (lead); data curation (lead); formal analysis (lead); funding acquisition (lead); investigation (lead); methodology (lead); project administration (lead); writing – original draft (lead); writing – review and editing (lead). **Fei Liu:** Conceptualization (equal); data curation (equal); formal analysis (equal); writing – review and editing (equal). **Wen‐Jie Ma:** Supervision (equal); validation (equal); visualization (equal). **Hai‐Jie Hu:** Supervision (equal); validation (equal); visualization (equal). **Yan‐Wen Jin:** Supervision (equal); validation (equal); visualization (equal). **Fu‐Yu Li:** Writing – review and editing (equal).

## FUNDING INFORMATION

This study was supported by 1.3.5 project for disciplines of excellence, West China Hospital, Sichuan University (ZYJC21046); 1.3.5 project for disciplines of excellence‐Clinical Research Incubation Project, West China Hospital, Sichuan University (2021HXFH001); Natural Science Foundation of Sichuan Province (2022NSFSC0806); National Natural Science Foundation of China for Young Scientists Fund (82203650, 82203782), Sichuan Science and Technology Program (2021YJ0132, 2021YFS0100); The fellowship of China Postdoctoral Science Foundation (2021M692277); Sichuan University‐Zigong School‐local Cooperation project (2021CDZG‐23); Sichuan University‐Sui Lin School‐local Cooperation project (2022CDSN‐18); Science and Technology project of the Health planning committee of Sichuan (21PJ046); Post‐Doctor Research Project, West China Hospital, Sichuan University (2020HXBH127). Funding source has no role in design and preparation of the manuscript.

## CONFLICT OF INTEREST STATEMENT

All authors declare having no conflicts of interest to disclose.

## Data Availability

All data generated or analyzed during this study came from our cancer database and can be provided if required.
